# MStargetR: a reproducible, containerised workflow for end-to-end targeted (MRM/SRM) mass spectrometry data processing in R

**DOI:** 10.1007/s11306-026-02541-2

**Published:** 2026-09-25

**Authors:** Harrison Szemray, Vimalnath Nambiar, Dana Hicks, Samantha Lodge, Julien Wist, Nathan G. Lawler, Luke Whiley

**Affiliations:** 1 Centre for Computational and Systems Medicine, Health Futures Institute, Murdoch University, Perth, WA Australia; 2 School of Medical, Molecular, and Forensic Science, Murdoch University, Perth, WA Australia; 3 School of Biomedical Sciences, University of Western Australia, Perth, WA 6009 Australia; 4 School of Allied Health, College of Health and Education, Murdoch University, Perth, WA Australia; 5 Chemistry Department, Universidad del Valle, Cali, 76001 Colombia; 6 Curtin Medical Research Institute, Curtin University, Perth, WA Australia; 7 School of Diagnostic and Therapeutic Sciences, Curtin University, Perth, WA Australia; 8 Dementia Centre of Excellence, Enable Institute, Curtin University, Perth, WA Australia

**Keywords:** Precision medicine, Phenotype, Lipidomics, Mass spectrometry, Multiple reaction monitoring, Reproducibility of results, Software

## Abstract

**Introduction:**

Targeted metabolic phenotyping by liquid chromatography–tandem mass spectrometry (LC-MS/MS) relies on a fragmented toolchain of proprietary vendor formats, manual integration steps, and ad hoc quality-control (QC) scripts, introducing user- and laboratory-level variation that undermines reproducibility and confounds cross-laboratory and retrospective comparison.

**Objectives:**

To provide an open-source, R-native workflow for targeted multiple reaction monitoring (MRM/SRM) mass spectrometry data that consolidates vendor file conversion, peak integration, and QC reporting into a single reproducible pipeline while preserving auditable, human-in-the-loop peak review.

**Methods:**

MStargetR builds on msConvert and Skyline through three modules: msConvertR (vendor-to-mzML conversion), PeakForgeR (peak boundary optimisation and automated peak integration executed through Skyline), and qcCheckR (normalisation, concentration calculation, signal and batch correction, and reporting). Additionally, MStargetR has a standalone correction module and a Shiny graphical user interface. Third-party tools are pinned in version-controlled Docker images (with Apptainer support for high-performance computing), and each analytical plate emits a fully populated sky document for inspection and reimport.

**Results:**

Applied to a published targeted lipidomics dataset of 128 human plasma samples targeting 1,161 lipid species, MStargetR processed all samples end-to-end, recovering all 1,161 targeted lipid features, 949 of which (81.7%) were detected and returned RSD < 30% across replicated long-term reference QCs. Analysis scaled linearly to 4,200 samples, averaging 4.1 s per sample.

**Conclusion:**

MStargetR delivers automated batch processing, auditable peak review, and a documented QC layer in a single reproducible pipeline, supporting FAIR-aligned targeted metabolomics.

**Supplementary Information:**

The online version contains supplementary material available at https://doi.org/10.1007/s11306-026-02541-2.

## Introduction

Targeted metabolic phenotyping, which uses liquid chromatography tandem mass spectrometry (LC-MS/MS) to quantify selected metabolites across diverse biological matrices such as blood, urine, and saliva, has become a routine tool in clinical research (Anh et al., 2024; Ryan et al., 2023). Targeted methods can offer increased sensitivity, accuracy, and quantitative reliability. This precision makes targeted phenotyping ideal for use in hypothesis-driven studies, biomarker validation, and mechanistic investigation of metabolic pathways (Roberts et al., 2012). Applications span a wide range of disciplines, including personalised medicine (Ruffieux et al., 2023), pharmacology (Deschamps et al., 2023), and nutritional science (LeVatte et al., 2022), where accurate metabolite measurements are critical for linking biochemical changes to physiological outcomes (Zhou & Zhong, 2022). Furthermore, the integration of targeted metabolic phenotypes with other omics data, such as genomics and proteomics, known as multiomics, has accelerated systems biology approaches, enabling comprehensive characterisation of metabolic networks and their perturbations in health and disease (Hayes et al., 2024). Targeted metabolic phenotyping continues to serve as a cornerstone analytical approach for biomarker validation, modelling of biological conditions and disease, and pathway elucidation (Zhou & Zhong, 2022).

Processing targeted LC-MS/MS data remains a practical bottleneck for most research groups. Vendor-specific software creates pipeline dependencies that complicate cross-laboratory harmonisation, and proprietary data formats restrict long-term accessibility and FAIR-aligned reuse, making conversion to open formats such as mzML (Martens et al., 2011) a necessary first step. Beyond format conversion, metabolic phenotyping experiments are susceptible to systematic variation arising from sample preparation, instrument performance, and batch effects (Hajnajafi & Iqbal, 2025), and the choice of processing tool and parameterisation itself introduces additional variance (Wartmann et al., 2024). Robust, standardised QC procedures are therefore essential for ensuring result validity and enabling reliable retrospective comparisons.

Despite the importance of targeted workflows, the development of metabolomics software has favoured untargeted ecosystems. Tools such as XCMS (Tautenhahn et al., 2012), MZmine3 without a paid subscription (Schmid et al., 2023), and ADAP-big (Du et al., 2020) deliver mature, end-to-end pipelines for feature detection, alignment, and annotation, but are not designed for the transition list-driven quantification that defines targeted MRM/SRM workflows. In the targeted space, researchers have largely depended on proprietary vendor environments, open-source MRM processors, or Skyline (Adams et al., 2020), each of which addresses part of the problem but leaves a critical gap.

Vendor platforms such as SCIEX OS, Agilent MassHunter Quantitative Analysis, and Waters TargetLynx provide rich peak inspection and manual integration. However, they are platform-locked, licence-dependent, and tied to a single instrument vendor’s data format, restricting cross-laboratory portability and FAIR-aligned data sharing. A number of dedicated open-source MRM processors have been developed. MRMPROBS provides a Windows graphical environment that imports four major vendor formats and mzML and performs probabilistic peak identification, quantification, and QC-sample-based normalisation for large MRM assays (Tsugawa et al., 2014). automRm is an R package that applies machine learning to peak picking and to the classification of peak quality in LC-QQQ-MS data, operating on mzML files that the user must convert beforehand (Eilertz et al., 2022). MRM Processor combines relative retention time correction with derivative-based signal characterisation and calibration-based quantification (Chen et al., 2026), and MRMPro takes a complementary route, providing a browser-based, multi-user platform explicitly optimised for the speed of manual peak inspection in large cohorts (Wang et al., 2024). MRMQuant automates quantitation for large-scale targeted studies (Lynn et al., 2024). Skyline provides an open, vendor-neutral environment for visualising chromatograms and refining boundaries (Adams et al., 2020), but is a GUI-driven application that does not, on its own, deliver a scriptable, R-native batch workflow with bundled QC. These tools variously offer automation, interactive review, or both, but none of them combines vendor file conversion, automated integration, a re-importable artefact that carries every chromatogram and boundary behind a reported value, and a documented QC and batch correction layer within a single, scriptable, version-pinned environment. The gap, therefore, is an open-source, R-native pipeline that combines automated batch processing with the auditability of Skyline, paired with a documented and configurable QC layer.

To address this gap, we present MStargetR, an open-source, R-native workflow that provides infrastructure for targeted MRM/SRM metabolic phenotyping, built on the established community tools ProteoWizard msConvert (Adusumilli & Mallick, 2017) and Skyline rather than replacing them. The package comprises three core modules, each standardising one stage of the pipeline: msConvertR converts proprietary vendor files to open mzML format; PeakForgeR performs retention time alignment, peak boundary optimisation, and integration via Skyline’s command-line interface; and qcCheckR handles missing value imputation, internal standard normalisation, concentration calculation, signal drift and batch correction, filtering, and reporting. A standalone batch correction module exposes the correction routines for use on external datasets. A Shiny-based graphical user interface provides code-free access to the full pipeline, retaining the same containerised execution as the programmatic workflow. The third-party tools are pinned to versioned Docker images, with Apptainer support for high-performance computing environments, eliminating complex dependency management and ensuring reproducible execution natively across AMD64 infrastructure on Windows and Linux operating systems. Users on macOS or ARM64 devices will need to run the pipeline inside a virtual machine. Critically, PeakForgeR emits a fully populated.sky document per analytical plate that can be reopened in Skyline for inspection, manually corrected, and reimported by qcCheckR without rerunning upstream steps, preserving the human-in-the-loop peak review that analysts rely on while delivering fully automated batch processing.

## Methods and implementation

**Fig. 1:**
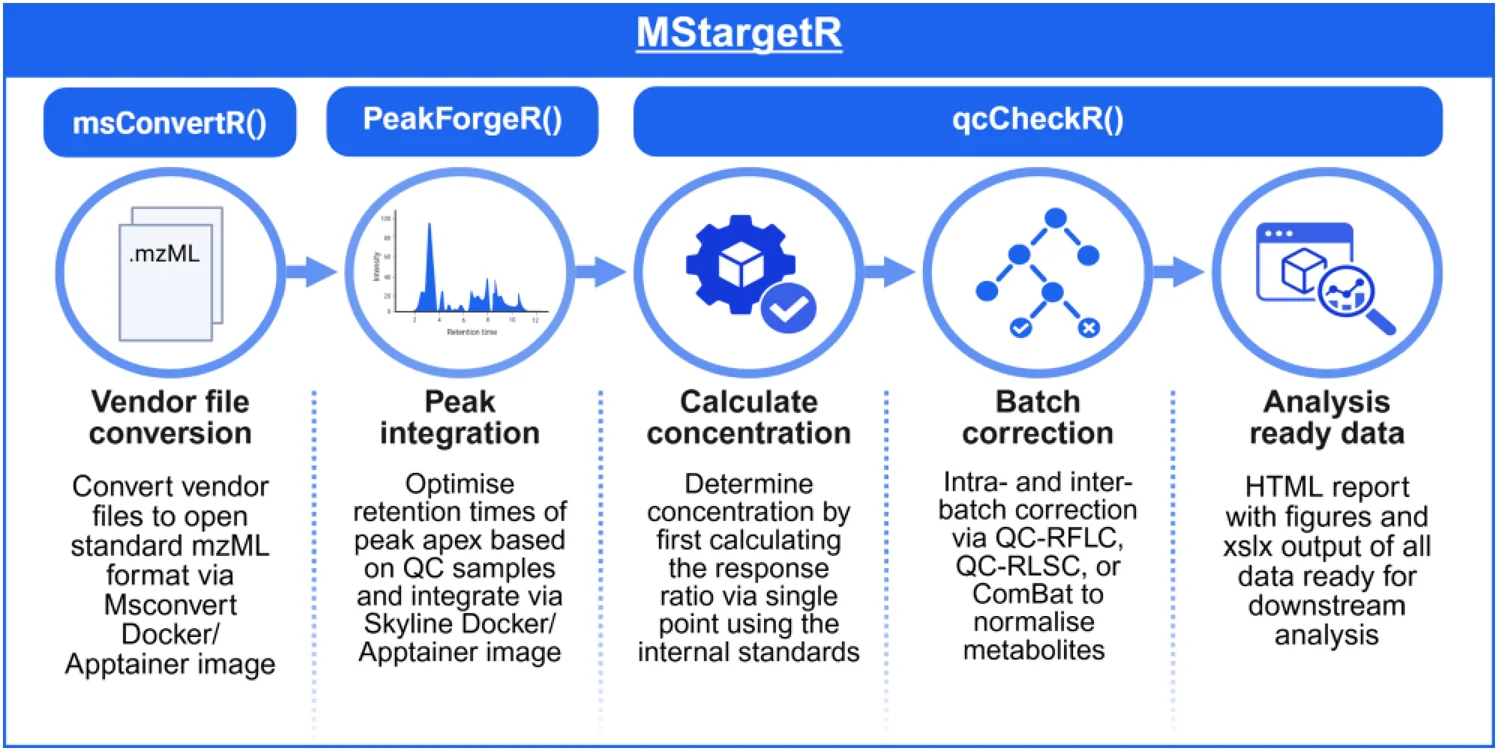
Graphical illustration of the core workflow of the MStargetR package. Created in BioRender. Szemray (2026) https://BioRender.com/n03kht2

MStargetR is implemented as an R package with a modular design in which three core functions address sequential stages of the targeted metabolomics processing workflow (Fig. 1). To ensure computational reproducibility and eliminate complex dependency management, all third-party tools are bundled within version-pinned Docker images (Boettiger, 2015), encapsulating msConvert by Proteowizard (Adusumilli & Mallick, 2017) and Skyline by the MacCoss Lab (Adams et al., 2020). This containerised approach enables consistent execution across operating systems with AMD64 architecture and removes complex dependencies and setup. For users operating in high-performance computing environments that prohibit Docker, MStargetR can be configured to leverage Apptainer (formerly Singularity). To achieve scalability, MStargetR implements parallelised execution across analytical batches, leveraging concurrent processing to optimise throughput.

For readers who are less familiar with these components, R is the environment in which the user, or the graphical interface on the user’s behalf, issues commands, and in which all data handling, retention time alignment, quality control, and report generation are performed. Docker, or Apptainer in high-performance computing environments, is a lightweight virtualisation layer that ships msConvert and Skyline together with the exact versions of their dependencies as a pre-built image, so that the user never has to install or configure either tool. msConvert and Skyline are then executed non-interactively inside that image through their command-line interfaces, invoked by MStargetR and returning their outputs to R. Shiny provides an optional browser-based front end that issues the same R calls through menus and file selectors instead of code. From the user’s point of view, therefore, a single function call, or a single click in the interface, triggers a fully specified sequence of containerised operations, with the mzML files, the per-plate.sky documents, and the QC reports appearing in the project directory as the visible outputs. A user guide written for analysts without prior R experience is hosted at https://mstargetr.github.io/MStargetR/ and is also included in the repository. It covers installation on each supported operating system, a fully worked quick start using the example dataset, a reference page for every exported function stating its inputs and its outputs. To aid in understanding of this section please see supplementary materials for MStargetR architecture demonstrating core functions and the associated inputs and outputs (Supplementary Figure S1), as well as Supplementary Table S2 for a tabularised view of MStargetR functions.

### msConvertR: proprietary to open file conversion

The msConvertR function interfaces with Proteowizard’s msConvert tool (Adusumilli & Mallick, 2017), executed within a Docker/Apptainer container, to convert proprietary vendor files into mzML format, the HUPO proteomics standards initiative community standard (Martens et al., 2011). The msConvertR function accepts raw data in the vendor formats supported by the bundled ProteoWizard release, namely SCIEX (.wiff, supplied together with its paired.wiff.scan file, and.wiff2), Thermo (.raw), Agilent (.d), Bruker (.d) and Waters (.raw), as well as data already in the open mzML or mzXML formats. Agilent, Bruker and Waters data are directory-based formats rather than single files: the complete, unmodified acquisition folder must be supplied, with its.d or.raw extension intact, and not an individual file contained within it. This is the most common cause of conversion failure in practice, and it is the reason a Bruker.d acquisition will not convert if it has been unpacked, renamed, or partially copied. Additionally, there is no support for Bruker.d files from EVOQ instruments, this limitation extends to msConvertR and Skyline. Accepted inputs, the required directory layout, and guidance on the most frequent conversion errors are set out in Supplementary Table S3 and in the vendor format section of the online user guide. The converted mzML files are organised into a preestablished file structure within the project directory for downstream processing.

### PeakForgeR: peak boundary optimisation for automated integration

PeakForgeR first performs peak boundary optimisation, before guiding automated peak integration. To optimise memory usage and ensure scalability in multi-plate sample analyses, data is processed on a plate-by-plate batch basis. For each analytical batch, a lightweight algorithm first identifies the peak apex and peak boundaries for each transition window. This is performed using the median apex retention time localisation across QC samples to define a plate-wide reference. The resultant pre-defined boundaries are then automatically pushed to the Skyline runner command line interface to perform peak area integration within the specified boundaries. This ensures consistent integration windows and consistent peak processing rather than executing independent per-sample peak detection that may introduce variable integrals. By using skyline runner over an integration process within R itself, facilitates the production of.Sky files which then allow for manual peak review inside the Skyline GUI if required. The production of.sky files also allows for data sharing of peak integration outputs in non-proprietary data format.

It should be noted that PeakForgeR uses Skyline runner as an interface tool in peak integration because of the capability to produce.Sky files. It does not actively invoke Skyline peak finding algorithms, although this can be done manually by the user post-hoc if desired. The.sky document preserves every chromatogram, retention time annotation, and peak boundary used to produce the result table, and can be opened directly in Skyline by the analyst or any collaborator with access to the file. Individual peaks can be visually inspected, integration boundaries can be adjusted where automated detection has not met user expectations, and the corrected results can be re-exported and reimported by qcCheckR without re-executing msConvertR or the upstream PeakForgeR steps. This handoff turns automated peak picking into a transparent first pass that the user remains in control of and turns the.sky document itself into a shareable, auditable record of every integration decision behind a reported concentration matrix. This feature aligns with current metabolomics QC reporting guidance and with FAIR data principles for processed analytical outputs.

Acquisition methods that alternate between positive and negative electrospray polarity within a single injection require no additional configuration. Polarity is recorded per spectrum in the mzML file written by msConvert and is carried through to Skyline, where each transition is matched to chromatogram data of the corresponding polarity using the sign of the precursor charge declared in the transition list. Mixed-polarity acquisitions therefore do not need to be split into separate files, and files that contain both positive and negative data are processed within the same analytical plate. Which transitions within the file are processed is determined entirely by the user-supplied transition list: every precursor (Q1) to product (Q3) pair in the list, including multiple product ions or qualifier transitions belonging to the same precursor, is extracted, aligned and integrated as an independent target, and a transition is included or excluded simply by editing that list. MStargetR is designed for MRM and SRM data; product ion spectra acquired in data-dependent or data-independent modes are outside the scope of the current release.

### qcCheckR: concentration calculation and quality control

The qcCheckR function implements QC procedures and generates analysis-ready data matrices and QC reports. The process takes the integrated peak areas from PeakForgeR, either from the initial automated run or after manual correction in Skyline and applies a series of QC steps. Missing values below the user specified instrumental limit of detection (e.g. peak area less than 5000) are handled using x_min_/2 (i.e. the minimum observed value for that specific feature), thereby maintaining data integrity (Wei et al., 2018). Metabolite concentrations are then calculated by first determining the response ratio (target metabolite peak area divided by the internal standard peak area) and multiplying it by the user-provided internal standard concentration factor. This method ensures that concentrations are anchored to a controlled point while correcting for systematic errors and improving overall accuracy. To account for systematic variations, the function offers three popular methods to correct for batch effects and signal drift: quality control sample based random forest signal correction (QC-RFSC) (Luan et al., 2018) quality control based robust locally estimated scatterplot smoothing (LOESS) signal correction (QC-RLSC) (Lin & Dunn, 2025), and ComBat (Zhang et al., 2020), allowing laboratories to decide which correction method best fits their own practices. The qcCheckR function also offers flexible filtering options, enabling users first to select which QC samples to utilise based on naming conventions, then exclude samples or features based on customisable quality metrics, such as the percentage of missing data or the relative standard deviation observed in selected QC samples.

We recommend that researchers include both long-term reference samples and pooled quality-control samples throughout the analytical run. Examples include pooled QC samples for quality control within a single study, or long-term reference samples (LTR) to enable reliable comparisons across different studies (Broadhurst et al., 2018; Grant-St James et al., 2025). qcCheckR generates comprehensive outputs in both HTML and Excel formats. The HTML report includes summary tables highlighting key QC metrics, and interactive visualisations such as PCA plots and control charts that allow dynamic exploration of data quality. The Excel output contains the data and metadata produced at each step of the MStargetR pipeline. Together, these outputs enable researchers to assess data quality and proceed with downstream statistical analyses, while the per plate.sky documents produced upstream remain available as the visual, auditable basis for every reported value.

### batchCorrectR: standalone inter- and intra-batch correction

The batchCorrectR function exposes the signal drift and batch correction routines of the MStargetR pipeline as a standalone module, allowing users to apply correction to any tabular metabolomics dataset independently of the upstream workflow. The function accepts a data frame, or a list of data frames to be combined, in which samples are arranged as rows and metabolite features as columns, requiring only the columns that identify the sample name, analytical batch, sample type, and run order. This design makes batchCorrectR suitable for data that have already been processed through other software, or that originate outside the MStargetR workflow, or for users wishing to apply a secondary correction.

Correction can be performed via three methods. QC-RFSC, which fits a random forest to the QC samples as a function of injection order within each analytical batch and applies the learned correction to every sample, capturing non-linear drift that linear models cannot (Luan et al., 2018). Quality control injections whose total signal falls below 10% of the batch median are flagged and reclassified as study samples so that failed injections do not distort the correction model, and corrected values are subsequently rescaled so that QC means match their pre-correction magnitude, preserving the absolute scale of the reported concentrations. Alternatively, QC-RLSC can be applied depending on study size and user preference (Lin & Dunn, 2025). For datasets that lack QC samples, batchCorrectR instead offers ComBat, an empirical Bayes method that estimates and removes inter-batch location and scale effects directly from the batch structure (Zhang et al., 2020). The function returns the corrected data matrix together with per metabolite relative standard deviations (RSD) before and after correction and, optionally, an HTML report and diagnostic visualisations including run order, principal component analysis, and RSD comparison plots.

### launchMStargetR: a graphical interface to the full pipeline

**Fig. 2:**
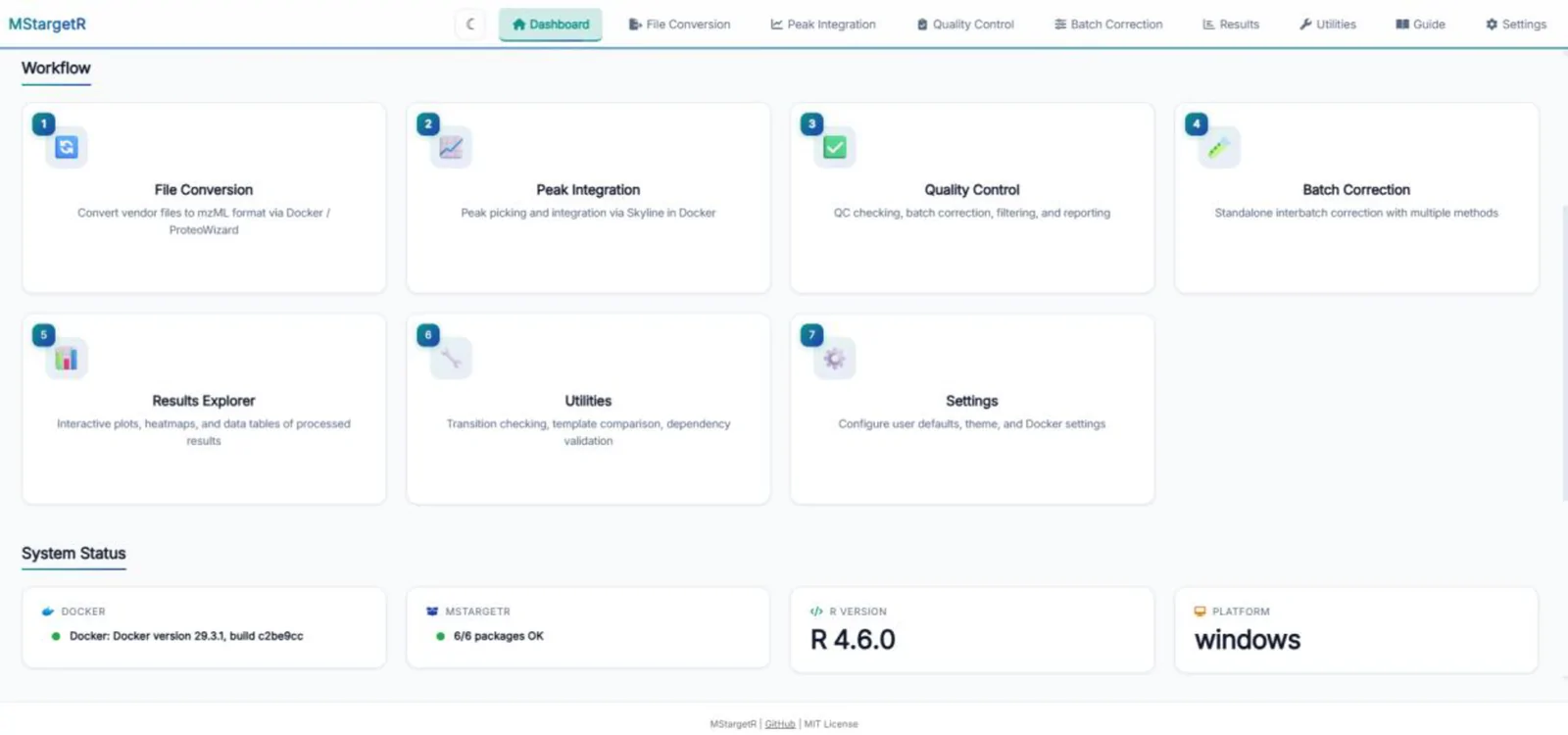
MStargetR graphical user interface. The application guides users through each stage of the MStargetR pipeline as a series of tabs, and supports interactive exploration and export of results

Users who prefer a graphical user interface can launch the entire MStargetR workflow from any R session by calling launchMStargetR, which opens a local interactive application built with Shiny (Chang et al., 2026) (Fig. 2). Alternatively, for users seeking a code-free experience, a Windows installer (.exe) can be downloaded from the latest GitHub release. The installer provides the graphical interface and its launcher directly, so that the user is not required to install, configure, or know anything about R, RStudio, or Shiny: a container runtime and the installer are the only two downloads, after which the application is started from the Start menu like any other Windows program. A step-by-step installation walkthrough for each supported operating system is provided in the online user guide (see Software and Data Availability). The application presents each stage of the pipeline as a guided tab, allowing the user to convert vendor files with msConvertR, integrate peaks with PeakForgeR, calculate concentrations and perform QC with qcCheckR, and apply standalone batch correction with batchCorrectR, all through file selectors and parameter fields rather than R code. Results are explored interactively within the same interface through quality dashboards, principal component analysis plots, run order and control charts, and per metabolite summaries, and can be exported as the same HTML and Excel reports produced by the programmatic workflow. The application is served locally by default and can optionally be exposed on a network for shared use, for which standard reverse proxy and authentication safeguards are recommended.

### Utility functions for transition list validation

Targeted analyses depend on a correctly specified transition list, and mistakes in this user provided table, duplicated precursor/product ion pairs, absent columns, or internal standards that are referenced inconsistently between files propagate silently into ambiguous peak assignments and incorrect concentrations. MStargetR therefore provides a small set of validation utilities that interrogate the transition list before it is used. These are exposed for the user to run interactively and are also invoked automatically within PeakForgeR and qcCheckR, so that a malformed list halts the workflow with an explicit, actionable message rather than producing misleading results. The transition_checkR function examines the multiple reaction monitoring (MRM) transition list. It confirms that every precursor (Q1) and product (Q3) ion mass to charge pair is unique within a configurable mass tolerance (0.001 Da by default, applied to avoid spurious clashes arising from the floating point parsing of the input file). In the event duplicate pairs are found, it returns the clashing transitions for the analyst to correct, since such clashes would otherwise generate ambiguous peak assignments downstream. The compare_mrm_template_with_guide function checks that every stable isotope-labelled (SIL) internal standard referenced in the transition list has a corresponding entry in the concentration guide, because an unmatched standard would cause the response-ratio concentration calculation in qcCheckR to fail.

Two further functions, validate_mrm_template_list and validate_qcCheckR_mrm_template_list, perform structural validation of the supplied templates: they confirm that the required columns are present, that any file paths resolve to readable.csv or.tsv files, and that critical fields, including ion mass-to-charge values, charges, and retention-time windows, contain no missing values before processing begins. These checks are deliberately conservative. They are designed to catch the common, easily overlooked errors in a hand-curated transition list rather than to verify the chemical correctness of the targets themselves, and by failing early with specific messages, they spare the user from discovering a formatting error only after a file conversion and integration run.

To evaluate MStargetR performance and demonstrate its practical utility, we utilised a publicly available targeted lipidomics dataset comprising 128 human plasma samples (Govus et al., 2025). Please refer to https://doi.org/10.6084/m9.figshare.31450306 for data availability and associated license (Creative Commons Attribution 4.0 International). The dataset consisted of 96 biological samples, 16 pooled QC samples that were made up of equal parts of each study sample, and 16 long term reference samples, which were a commercial pool external to the study used to monitor long term laboratory performance. The analytical method targeted 1,161 lipid species, using 73 stable isotope labelled internal standards for normalisation (Govus et al., 2025).

## Results and discussion

MStargetR implements a streamlined workflow requiring minimal configuration. Users organise raw vendor files into a project directory structure and sequentially execute three functions: msConvertR, PeakForgeR, and qcCheckR. The package generates a comprehensive output structure by analytical plate, with results organised into plate specific folders as well as a project level `ALL` folder that aggregates data from multiple vendor files. The structure includes the converted.mzML files, intermediate data from PeakForgeR (including the per plate.sky documents), and the final analysis ready outputs from qcCheckR.

The primary technical contribution of MStargetR is the consolidation of a fragmented, multi-step preprocessing pipeline into a unified, containerised environment that uses version pinned Docker images with the option to configure Apptainer for high-performance computing, bypassing Docker restrictions. This architectural choice bridges the gap between the high automation, low transparency environment of proprietary vendor software and the transparency of open source R packages, while explicitly preserving an inspectable handoff to Skyline. By emitting a.sky document at the end of PeakForgeR, MStargetR retains the visual, peak by peak inspectability that analysts depend on in proprietary tools but delivers it openly and cross platform.

### MStargetR performance evaluation

**Fig. 3:**
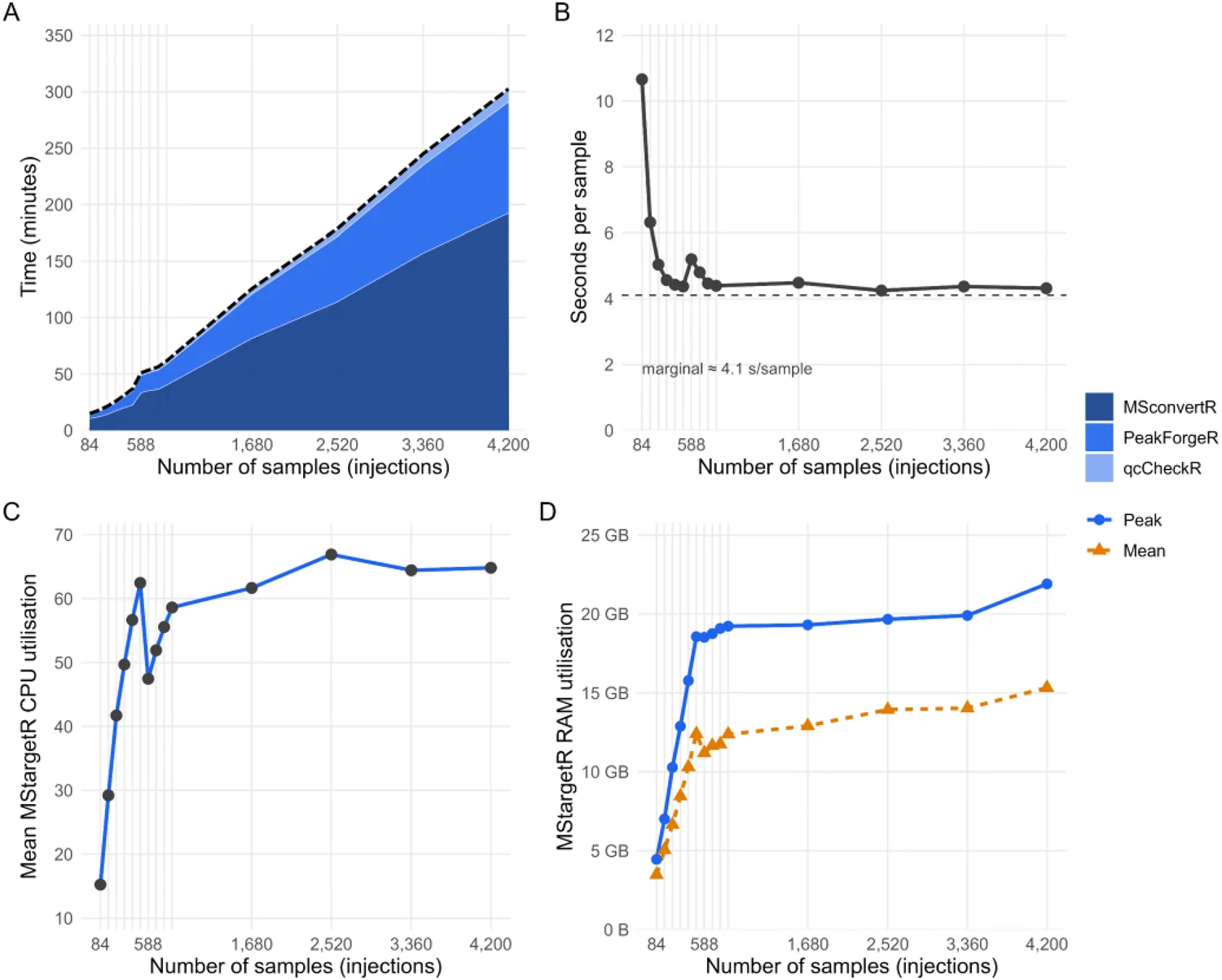
MStargetR pipeline scalability benchmark (84 − 4,200 samples). **A** Total wall-clock runtime (minutes) versus number of samples, split by pipeline step: msConvertR (dark blue), PeakForgeR (medium blue), and qcCheckR (light blue); dashed line shows a linear reference fitted to the largest run. **B** Per-sample processing time (seconds/sample) at each scale point; dashed line indicates the marginal cost (4.1 s/sample). **C** Mean MStargetR CPU utilisation (%). **D** Peak (blue) and mean (orange, dashed) MStargetR RAM consumption (GB). Created in R version 4.5.1 (2025-06-13 ucrt)

We performed a throughput test, using a workstation equipped with an 11th Gen Intel Core i7-11370 H CPU (3.30 GHz base clock, 4 cores, 8 logical processors), 40 GB of DDR4 RAM, and a 2 TB NVMe Samsung SSD 980, running Microsoft Windows 11 Home. The software was implemented in R version 4.5.1 (2025-06-13 ucrt). The throughput test consisted of processing increasing numbers of samples through the full MStargetR pipeline, as shown in Fig. 3, including conversion to mzML, chromatogram extraction, peak detection, data aggregation, quality checking, and reporting.

The full MStargetR pipeline scaled linearly with sample count across 84 to 4,200 injections (Fig. 3A). Total runtime grew from 14.9 min at 84 samples to 302.5 min (5.04 h) at 4,200 samples, closely tracking the linear reference throughout. msConvertR (63.7% of wall time) and PeakForgeR (32.6%) were the dominant cost drivers, with qcCheckR contributing a minor fraction (3.7%) at all scales. Per-sample throughput improved markedly with batch size: per-sample time fell from 10.7 s per sample at 84 injections to 4.1 s per sample at ≥ 840 injections (Fig. 3B), reflecting the disbursement of fixed per-run overhead across larger batches. System resource usage displayed two distinct scaling regimes. CPU utilisation (Fig. 3C) rose from 15.3% at the smallest batch (84 samples) to a plateau of approximately 62–67% system-wide above 1,680 samples, with little change at larger sample counts. Because parallelism was capped at (total cores − 2) of 8 logical cores, reserving 2 cores for system processes, system wide utilisation is bounded at approximately 75%, the observed plateau sits just below this ceiling, indicating that the worker cores run near full occupancy once enough plates are present to keep them supplied. RAM consumption (Fig. 3D) followed a similar two phase pattern. Peak RAM climbed sharply from 4.46 GB (84 samples) through a successive number of samples, reaching 19.23 GB by 840 samples, then grew to 21.91 GB at 4,200 samples. This memory behaviour is consistent with a footprint dominated by the per plate parallelism overhead rather than per sample data accumulation. Together, these results indicate that total analysis time scales approximately linearly with sample count, with low and stable marginal cost per sample, supporting predictable performance for large batch runs.

### Case study: analysis of a human performance plasma sample set

**Fig. 4:**
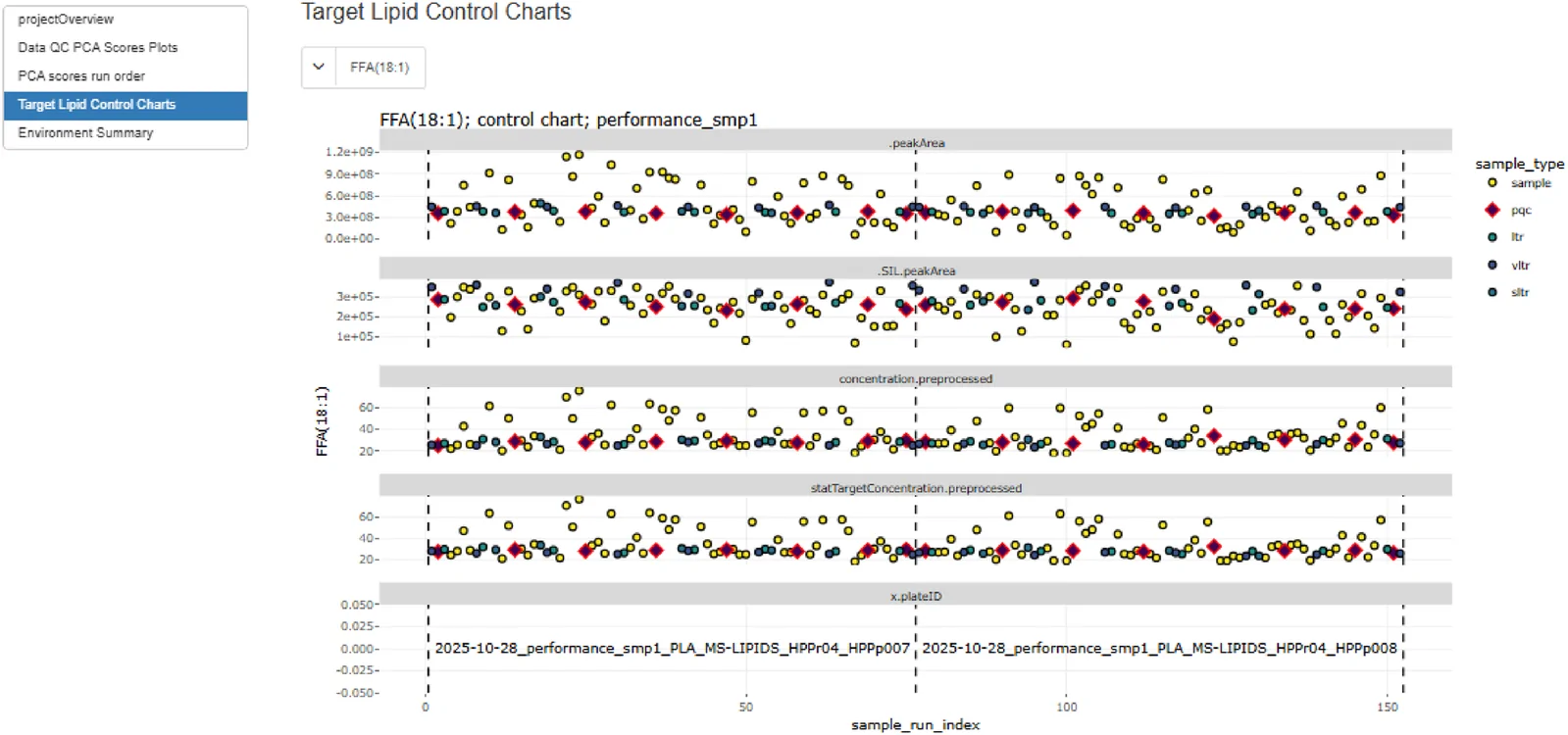
Snapshot of the interactive qcCheckR HTML quality control report. The target lipid control charts view is shown for a representative lipid species (FFA(18:1)), with panels (top to bottom) for raw peak area, internal-standard (SIL) peak area, preprocessed concentration, and batch/signal drift corrected concentration, plotted against sample run order and coloured by sample type

To demonstrate its practical utility, we processed a publicly available targeted lipidomics dataset comprising 128 human plasma samples (Govus et al., 2025). The dataset consisted of 96 biological samples, 16 pooled QC samples that were made up of equal parts of each study sample, and 16 long term reference samples, which were a commercial pool external to the study used to monitor long term laboratory performance (Govus et al., 2025). The analytical method targeted 1,161 lipid species, using 73 stable isotope labelled internal standards for normalisation. Quality control metrics indicated robust analytical performance: of the 1,161 detected lipid features, 949 lipid features (81.7%) had a relative standard deviation (RSD) < 30% for peak area across long term reference QC samples, and 962 features met this threshold when concentrations were calculated (i.e., single point using the internal standards). When using stringent filtering (i.e. QC RSD < 10%), 823 features were suitable for statistical analysis. These figures are analytical precision thresholds applied across the full 1,161-transition panel, and should not be read as detection rates. The 81.7% therefore reflects the combined effect of biological absence, method sensitivity, and the deliberately stringent RSD < 30% criterion applied to long-term reference samples, rather than a failure of peak detection. Because the per-transition relative standard deviations and the corresponding chromatograms are both retained, in the qcCheckR report and in the per-plate.sky documents respectively, the reason any individual target falls below the threshold can be inspected directly rather than inferred. In this dataset, no samples or internal standards were flagged for missing values (i.e., missing values in more than 50% of samples). To visualise the data quality, principal components analysis revealed apparent clustering by sample type and plate ID, indicating minimal batch effects. The final QC report was generated in both HTML and Excel formats, including interactive visualisations and control charts for key lipid targets (Fig. 4). These control charts plot each target across sample run order, allowing analytical stability and the effect of successive normalisation steps to be inspected at the level of individual lipid species. Resources required to reproduce this case study and example outputs are listed in the Availability section.

The qcCheckR control charts provide a per-target view of analytical performance across the analytical run. For each specified control lipid species, the response is plotted against sample run order and coloured by sample type, so that biological samples, pooled QC, and long-term reference samples can be distinguished immediately. The stacked panels trace the data through successive processing stages: raw peak area, internal-standard (SIL) peak area, preprocessed concentration, and batch/signal drift corrected concentration, allowing the contribution of each normalisation step to be assessed directly. Tight, run-order-stable clustering of the QC and reference samples indicates consistent instrument response, whereas systematic drift, step changes at plate boundaries, or dispersion among QC replicates would be immediately apparent. The accompanying plate identifier panel anchors each observation to its acquisition batch, making it straightforward to attribute any trend to a specific plate or segment. For the representative species shown in Fig. 4 (FFA(18:1)), the QC and reference samples remain stable across the run, and the correction step further tightens their distribution, consistent with the minimal batch effects observed in the principal components analysis.

Taken together, the performance evaluation and case study results demonstrate that MStargetR performs robustly on a publicly available targeted lipidomics dataset, recovering many features within accepted quality thresholds, processing samples at throughput suitable for routine use, and generating auditable, analysis-ready outputs without manual intervention at any pipeline stage. The linear scaling behaviour further indicates that performance is predictable as sample numbers increase, allowing processing time for larger studies to be estimated reliably from the per-sample rate reported here. Consistent feature recovery, deterministic and scalable throughput, and fully automated report generation position MStargetR as a practical solution for high-throughput targeted metabolomics and lipidomics, where analytical rigour and reproducibility must be maintained across increasingly large and complex sample sets.

### Feature comparison to existing MRM tools

Table 1 provides a head-to-head comparison of MStargetR alongside Skyline and the dedicated open-source MRM processors MRMPROBS, automRm, MRM Processor, and MRMPro across fourteen features spanning platform and access, processing, inspectability and manual review, quantification and quality control, and reproducibility and scale. Read feature by feature, the processing core is common ground: every tool in the comparison performs automated peak boundary detection and integration, and all provide at least partial retention time correction. The differences concentrate at the two ends of the workflow. Upstream, automRm shares the R-native scriptability of MStargetR but operates on mzML files that the user must convert beforehand, whereas MStargetR bundles the converter within the pipeline so that raw multi-vendor data can be taken from conversion through processing to reporting in a single scripted call. Downstream, the layers that turn integrated peaks into an analysis-ready concentration matrix are the least served elsewhere: automated QC reporting is at best partial in the other tools, and signal drift and batch correction, which MStargetR provides with three selectable methods, is at most partial in every other tool in the comparison. MStargetR is also alone in exposing this correction layer as a standalone function (batchCorrectR) that can be applied to externally generated datasets.

The remaining rows concern reproducibility and auditability rather than algorithms. MStargetR is the only tool in the comparison distributed as a version-pinned container, and the only one with documented large-batch scaling at the level of thousands of samples (4,200 samples), where the other tools report at most cohort-scale validation. Together with Skyline, and uniquely among the dedicated processors, it also emits a fully re-importable artefact, a per-plate.sky document that preserves every chromatogram and integration boundary behind each reported value, so that any result can be audited and manually corrected without reprocessing the batch. The table equally records where MStargetR does not lead: quantification is performed by single-point internal standard calculation without multi-point calibration curves, an area in which the dedicated MRM processors are more complete, and MRMPro remains the stronger choice where rapid, multi-user manual curation of very large cohorts is the primary requirement. The contribution of MStargetR is therefore not any single algorithm but the consolidation of vendor file conversion, automated integration, full auditability, QC reporting, and batch correction within one open-source, R-native, version-pinned environment, a combination that Table 1 indicates no existing tool provides.

**Table 1:** Feature comparison of MStargetR with existing software for targeted (MRM/SRM) LC-MS data processing

Feature	MStargetR	Skyline	MRMPROBS	automRm	MRM Processor	MRMPro
*Platform and access*						
Open source	Yes (R package)	Yes (Apache 2.0)	Partial (free binary)	Yes (R package)	Yes (GPL-3.0, Python)	Partial (free web service)
Interface	R pkg + GUI + installer	Desktop GUI + CLI	Desktop GUI	R pkg + GUI	Python GUI	Web browser
Scriptable batch execution from R	Yes	Partial (CLI, not R-native)	No	Yes	Partial (Python scripting; not R)	No
Multi-vendor input with built-in conversion	Yes (bundled msConvert)	Yes (native readers)	Yes (ABF conversion)	No (mzML only)	No (mzML only)	Partial (AirdPro step)
*Processing*						
RT alignment/correction	Yes (QC-median apex)	Yes	Partial	Yes	Yes (RRT; core)	Yes
Automated boundary detection and integration	Yes	Yes	Yes	Yes	Yes	Yes
*Inspectability and manual review*						
Re-importable chromatogram + boundary artefact	Yes (per-plate.sky)	Yes (.sky)	Partial	Partial	No (summary + plot outputs)	Partial (cloud)
Manual correction without reprocessing	Yes	Yes	Yes	Partial	Partial (Excel-supplied ranges; rerun required)	Yes
*Quantification and QC*						
IS normalisation and quantification	Partial (single-point)	Yes	Yes	Partial	Yes	Yes
Drift/batch correction	Yes (3 methods)	No	Partial	No	No (RT only)	No
Automated QC reporting	Yes (HTML + Excel)	Partial (external)	Partial	Partial	No	No
Standalone correction of external data	Yes (batchCorrectR)	No	No	No	No	No
*Reproducibility and scale*						
Version-pinned container	Yes	No	No	No	No	No
Documented large-batch scaling	Yes (to 4,200 samples)	Partial	Partial	No	Partial (100-sample cohort validated)	Partial

Entries are Yes, Partial, No, or NR (not reported in the cited publication or documentation). Features were compiled from each tool’s primary publication and, where available, its documentation. This table should be read as a statement of scope and capability, and not as a claim of superior quantitative accuracy relative to the dedicated MRM processors

Abbreviations: CLI, command-line interface; IS, internal standard; NR, not reported; RRT, relative retention time. References: Skyline (Adams et al., 2020), MRMPROBS (Tsugawa et al., 2014), automRm (Eilertz et al., 2022) and MRM Processor (Chen et al., 2026) deliver automation, and MRMPro (Wang et al., 2024)

## Conclusions

Foundation metabolomics tools such as XCMS (Tautenhahn et al., 2012), MZmine (Schmid et al., 2023), MetaboAnalyst (Pang et al., 2024), and Skyline (Adams et al., 2020) have become community infrastructure through a combination of robust algorithms and, critically, their consolidation of complex workflows into accessible, standardised pipelines. MStargetR operates at this second level, building on the established capabilities of msConvert and Skyline to deliver something the targeted metabolomics community currently lacks: a single, open-source, R-native pipeline combining automated batch processing, auditable peak review, and a documented QC layer for targeted metabolomics pipelines. Proprietary vendor platforms provide rich visual inspection but are impaired by platform, licence, and format, restricting cross-laboratory portability and FAIR-aligned data sharing. Skyline addresses the open inspection problem and is the foundation on which PeakForgeR is built. Open-source MRM processors such as MRMQuant (Lynn et al., 2024), automRm (Eilertz et al., 2022) and MRM Processor (Chen et al., 2026) deliver automation, and MRMPro (Wang et al., 2024) provides a collaborative environment built for rapid manual review, but none of them returns to the analyst a single, self-contained, re-importable project file carrying every chromatogram and every boundary behind a reported value. MStargetR preserves the inspectability of a GUI workflow via per-plate.sky documents while delivering scriptability, version-pinned containerisation, and FAIR-aligned outputs. Its central design choice that leverages Skyline command line interface with resultant.sky file outputs, ensures automation does not come at the cost of the visual peak inspection analysts rely on. This particular combination is, to our knowledge, not currently offered by a single open-source package in the targeted metabolomics ecosystem. We would emphasise that the contribution lies in this integration and in the reproducibility it affords, rather than in any claim to superior peak detection accuracy over the dedicated MRM processors now available. As the metabolic phenotyping field continues to grow, a reproducible workflow infrastructure of this kind will become increasingly important for enabling researchers to focus on the biological questions that matter.

### Limitations and future work

At the time of publication, the Skyline back end used by PeakForgeR is not natively supported on devices with ARM64 architectures (e.g., Apple Silicon or Qualcomm Snapdragon X Elite/Plus processors) owing to architectural constraints of the underlying container; currently, this can be overcome by emulating a Windows AMD64 virtual machine on such devices, and we are actively exploring native solutions. Although MStargetR exposes Skyline’s peak inspection through the sky document handoff, large transition lists may still require analyst time for review on complex datasets; this is a property of any quantitative targeted workflow rather than of MStargetR specifically, although this process is aided through the transition list utility functions provided by MStargetR. Quantification in the current release is performed using single-point calibration, in which concentrations are derived from the response ratio of each target to its internal standard rather than from a multi-point calibration curve; this is well suited to the QC-anchored comparisons demonstrated here, but does not capture non-linear detector response across a wide concentration range. Bruker.d files acquired on Bruker QQQ systems (e.g. EVOQ) are not compatible with the package. This is not isolated to MStargetR, but also msConvert and Skyline as there is no supporting Bruker library available to enable conversion to mzML. Future work will focus on expanding the range of supported input formats, adding support for full multi-point calibration curves to enable absolute quantification across broader dynamic ranges, incorporating additional QC metrics and automated outlier detection, and exploring tighter integration with study design and reporting templates so that the sky documents and qcCheckR outputs can be deposited together in public repositories with minimal further effort.

## Supplementary Information

Below is the link to the electronic supplementary material.

Supplementary material 1 (PDF 169.2 kb)

Supplementary material 2 (DOCX 546.9 kb)

## Data Availability

Software and Data Availability: MStargetR is an open source R package with complete documentation and setup guide publicly available on GitHub at https://github.com/MStargetR/MStargetR. Docker image references and scripts are provided in the repository. The MStargetR codebase is also published on Zenodo (https://doi.org/10.5281/zenodo.18476537). Example inputs and outputs, along with a minimal working project resources to recreate the case study in the results section, are available at https://doi.org/10.6084/m9.figshare.31573066. To evaluate MStargetR performance and demonstrate its practical utility, we utilised a publicly available targeted lipidomics dataset comprising 128 human plasma samples (Govus et al., 2025). Please refer to https://doi.org/10.6084/m9.figshare.31450306 for data availability and associated license (Creative Commons Attribution 4.0 International).

## Abbreviations

AMD64: Advanced micro devices 64-bit architecture
ARM64: Advanced RISC machines 64-bit architecture
CPU: Central processing unit
CSV: Comma-separated values
DIA: Data-independent acquisition
FAIR: Findable, accessible, interoperable, reusable
GB: Gigabyte
HTML: HyperText markup language
HUPO-PSI: Human Proteome Organisation Proteomics Standards Initiative
IS: Internal standard
LC-MS: liquid chromatography-mass spectrometry
LOD: Limit of detection
MRM: Multiple reaction monitoring
MS: Mass spectrometry
MS/MS: Tandem mass spectrometry
mzML: Mass-to-charge markup language (open MS data standard)
QC: Quality control
QC-RFSC: Quality control based random forest signal correction
QC-RLSC: Quality control based robust LOESS signal correction
PC: Principal component
PCA: Principal components analysis
PRM: Parallel reaction monitoring
RSD: Relative standard deviation
SRM: Selected reaction monitoring
TSV: Tab-separated values
VM: Virtual machine
