# Supplementary material for: MStargetR: a reproducible, containerised workflow for end-to-end targeted (MRM/SRM) mass spectrometry data processing in R
Source: Metabolomics. 2026 Sep 25;22(5):160. doi: 10.1007/s11306-026-02541-2 (PMC13615125; doi:10.1007/s11306-026-02541-2)
Supplement: Supplementary file 1 [file 11306_2026_2541_MOESM1_ESM.pdf]

Minimum reporting standards document –
reporting the use of different QC samples in untargeted studies (version 1)

Authors: Metabolomics Quality Assurance & Quality Control Consortium (mQACC)
Community Engagement Working group

1. Please complete this form for each different analytical platform applied in the reported study. Different LC-MS assays can be grouped together in to a single form. Different NMR assays can be grouped together in to a single form.
2. If multiple different types of QC samples are used then list all in this form.
3. Definitions for different QC sample types is available at the end of the document

Questions

Q1. Which analytical platform(s) was applied in this study?

- NMR spectroscopy ☐
- GC-MS ☐
- LC-MS ☑
- CE-MS ☐
- DIMS ☐
- IR/Raman spectroscopy ☐
- Other ☐

Q2. Was a system suitability sample or suitability sample used during the reported study and is its composition and acceptance criteria reported in the manuscript?

YES ☐

NO ☐

NOT APPLICABLE ☑

Q3. Were internal standards used during the reported study and are the composition and acceptance criteria reported in the manuscript? For NMR, was an alternative calibration method, such as ERECTIC, used?

YES ☐

NO ☐

NOT APPLICABLE ☑

Q4. Were blank samples used during the reported study and are the composition and acceptance criteria reported in the manuscript?

YES ☐

NO ☑

Q5. Were one or multiple types of pooled QC samples used during the reported study and are the composition and acceptance criteria reported in the manuscript?

YES ☐

NO ☐

NOT APPLICABLE

Q6. Were sample collection, storage and thawing processes reported in the manuscript?

YES ☐

NO ☑

Q7. Were the order for sample preparation and data collection of biological samples randomised and is this reported in the manuscript?

YES ☐

NO ☑

Q8. Are the QC sample data available in a metabolomics data repository (e.g. MetaboLights or Metabolomics Workbench)?

YES ☐

NO ☐

NOT APPLICABLE ☑

Definitions

1. A ***system suitability test (SST)*** sample is a solution containing a small number of authentic chemical standards (typically five to ten analytes) from which the acquired data can be quickly assessed for accuracy and precision in an automated computational approach. For LC-MS and

GC-MS the analytes are dissolved in a chromatographically suitable diluent and not in a sample matrix.

2. ***Internal standards*** are compounds of predetermined concentrations and which are representative of the metabolite classes in the test sample metabolome and which are typically included in assays performed on mass spectrometry platforms. One or multiple internal standards are added to each test sample at the same concentration to allow monitoring of accuracy and precision for *m/z*, retention time, chromatographic peak shape, and peak area for every biological test sample.

3. Different types of ***blank samples*** can be analysed and include process/extraction blanks and solvent blanks. Process blank samples/extraction blank samples are samples which have passed through the sample preparation process in the same way as a biological sample but with no biological sample included. Solvent blank samples are samples which are composed of a solvent but which has not passed through the extraction process.

4. Different types of ***pooled QC samples*** can be analysed and include intra-study QC samples, intra-laboratory QC samples and inter-laboratory QC samples. Intra-study QC samples are prepared using biological samples analysed only in the study being reported. Intra-laboratory QC samples are pooled QC samples or a relevant material (e.g. a standard reference material) which are analysed in all studies within a single laboratory. Inter-laboratory QC samples are pooled QC samples or a relevant material (e.g. a standard reference material) which are analysed in single/all studies in different laboratories.
