# Supplementary material for: MStargetR: a reproducible, containerised workflow for end-to-end targeted (MRM/SRM) mass spectrometry data processing in R
Source: Metabolomics. 2026 Sep 25;22(5):160. doi: 10.1007/s11306-026-02541-2 (PMC13615125; doi:10.1007/s11306-026-02541-2)
Supplement: Supplementary file 2 [file 11306_2026_2541_MOESM2_ESM.docx]

**Supplementary Materials**

**MStargetR: A reproducible, containerised workflow for end-to-end targeted (MRM/SRM) mass spectrometry data processing in R**

Harrison Szemray^1,2^, Vimalnath Nambiar^1^, Dana Hicks^1,2^, Samantha Lodge^1^, Julien Wist^1,4^, Nathan Lawler ^1,3^, and Luke Whiley* ^1,5,6,7^

1. Centre for Computational and Systems Medicine, Health Futures Institute, Murdoch University, Perth, Western Australia, Australia
2. School of Medical, Molecular, and Forensic Science, Murdoch University, Perth, Western Australia, Australia
3. School of Allied Health, College of Health and Education, Murdoch University, Perth, Western Australia, Australia
4. Chemistry Department, Universidad del Valle, Cali, 76001, Colombia
5. Curtin Medical Research Institute, Curtin University, Perth, Western Australia, Australia
6. School of Diagnostic and Therapeutic Sciences, Curtin University, Perth, Western Australia, Australia
7. Dementia Centre of Excellence, Enable Institute, Curtin University, Perth, Western Australia, Australia

*correspondence: [luke.whiley@curtin.edu.au](mailto:luke.whiley@curtin.edu.au)

Author(s) ORCID:

Harrison Szemray: <https://orcid.org/0009-0008-5829-540X>,

Vimalnath Nambiar <https://orcid.org/0000-0001-5384-6788>,

Dana Hicks: <https://orcid.org/0000-0002-3823-8271>,

Samantha Lodge: <https://orcid.org/0000-0001-9193-0462>,

Julien Wist: <https://orcid.org/0000-0002-3416-2572>,

Nathan Lawler: <https://orcid.org/0000-0001-9649-425X>,

Luke Whiley: <https://orcid.org/0000-0002-9088-4799>.

**Supplementary Figure S1:** Overview of MStargetR architecture highlighting core functions as well as inputs and outputs.

**Supplementary Table S2**: Input and output overview for each exported MStargetR function. Functions are listed in the order in which they are used. Validation utilities return their result to the R session and do not write files; they are also invoked automatically inside PeakForgeR and qcCheckR, so that a malformed transition list halts the workflow with an explicit message.

| **Function** | **Stage** | **Input** | **Output written to the project directory** | **Returned to the R session** |
| --- | --- | --- | --- | --- |
| msConvertR | 1. Vendor file conversion | Project directory containing the raw vendor acquisition files (see Table S2); choice of container backend (Docker or Apptainer) | mzML files organised per analytical plate within the converted-data folder; conversion log | Summary of the files converted, with any msConvert errors reported per file |
| PeakForgeR | 2. Retention time alignment, boundary optimisation and integration | Project directory containing the mzML files from step 1; the MRM transition list (.csv or .tsv); QC sample naming pattern used to build the plate-wide retention time reference; retention time window settings | One folder per analytical plate containing: a fully populated .sky Skyline document (all chromatograms, retention time annotations and peak boundaries); the exported per-sample integrated peak areas; and a processing log | Summary of plates and transitions processed |
| (optional manual step) | Analyst review in Skyline | The per-plate .sky document produced by PeakForgeR, opened directly in Skyline | The corrected .sky document and its re-exported results table | Nothing; this step happens outside R and is optional |
| qcCheckR | 3. Concentration calculation, quality control and reporting | The integrated peak areas from PeakForgeR, either from the automated run or after manual correction in Skyline; the MRM transition list; the internal standard concentration guide (.csv); filtering thresholds (missing values, QC RSD); choice of correction method (QC-RFSC, QC-RLSC or ComBat) | A project-level ALL folder containing the HTML QC report (summary tables, PCA plots, per-target control charts) and a multi-sheet Excel workbook holding the data and metadata produced at every step of the pipeline | The analysis-ready concentration matrix together with the QC metrics used to produce the report |
| batchCorrectR | Standalone signal drift and batch correction | A data frame, or a list of data frames to be combined, with samples in rows and features in columns, containing columns that identify the sample name, the analytical batch, the sample type and the run order; choice of correction method | Optionally an HTML report with run order plots, PCA and a before/after RSD comparison | The corrected data matrix plus per-feature relative standard deviations before and after correction |
| launchMStargetR | Graphical interface to the whole pipeline | None at the call site; all paths and parameters are supplied interactively through the browser interface | The same outputs as the programmatic functions above | Starts a local Shiny application; results can be explored in the interface and exported as the same HTML and Excel reports |
| transition_checkR | Transition list validation | The MRM transition list; mass tolerance (0.001 Da by default) | None | The clashing transitions where any precursor (Q1) and product (Q3) pair is duplicated within tolerance, or confirmation that every pair is unique |
| compare_mrm_template_with_guide | Transition list validation | The MRM transition list; the internal standard concentration guide | None | Any stable isotope labelled internal standard that appears in one file but not the other, which would otherwise cause the response ratio calculation to fail |
| validate_mrm_template_list | Structural validation | The supplied MRM transition list template | None | Pass or fail, with explicit messages identifying missing required columns, file paths that do not resolve to a readable .csv or .tsv, and missing m/z, charge or retention time window values |
| validate_qcCheckR_mrm_template_list | Structural validation | The supplied qcCheckR template list | None | As above, for the templates consumed by qcCheckR |

**Supplementary Table S3** Input formats accepted by msConvertR, and the most common causes of conversion failure. Agilent, Bruker and Waters acquisitions are directory-based formats: the complete, unmodified acquisition folder must be supplied, with its extension intact, and not an individual file contained within it. Vendor reader coverage is provided by the ProteoWizard release pinned in the MStargetR container image; where a particular vendor build or acquisition mode is not covered by that release, the underlying msConvert message is written to the MStargetR log. For more information, please visit <https://mstargetr.github.io/MStargetR/articles/MStargetR-vignette.html>

| **Vendor** | **Extension** | **File or folder** | **What to supply** | **Most common cause of failure, and the fix** |
| --- | --- | --- | --- | --- |
| SCIEX | .wiff (with .wiff.scan), .wiff2 | File, with a paired companion file | The .wiff file with its matching .wiff.scan file present in the same directory | Conversion fails, or produces empty chromatograms, when the .wiff.scan file has not been copied alongside the .wiff. Copy both. |
| Thermo | .raw | Single file | The .raw file | Rarely problematic. Files still locked by the acquisition software will not be read; close the instrument software or copy the file first. |
| Agilent | .d | Folder | The entire .d folder, with the .d extension retained | Supplying a single file from inside the .d folder, or a folder whose .d extension has been stripped by an archiving tool. Supply the whole folder. |
| Bruker | .d | Folder | The entire .d folder, unmodified, with the .d extension retained | The most frequent failure in the pipeline. MSconvert ships no support for Bruker EVOQ data, so the files are unable to be converted to mzML format causing failure in pipeline. Additional causes are: the .d folder was unzipped, renamed, or only partially copied; an internal file (for example analysis.baf or analysis.tdf) was supplied instead of the folder; or the acquisition mode is not covered by the pinned ProteoWizard release. Supply the complete folder first, then check the MStargetR log for the msConvert message. |
| Waters | .raw | Folder | The entire .raw folder, with the .raw extension retained | As for Agilent: an individual file from inside the folder will not convert. |
| Any vendor, already converted | .mzML, .mzXML | Single file | The converted file, placed directly into the converted-data folder of the project directory | msConvertR is simply skipped in this case; PeakForgeR is run first instead. |
